# 

**Published:** 1885-01

**Authors:** 


					﻿JJkJNnj-JkRY, 1885.
OLD SERIES
vd. xxv.
NEW SERIESl
Vol. I. No. 11/ J
■X.	____ 1
& 1855 O ■’W
MwJ
Hi
M
EDIGAL,:
Surgical
40URNAW
L</4'by* W.F.WESTMORELANDjIf
H.V.M.MILLER,M.D.LL.D.>
tfV'’	JAMES A.GRAY, M. D.'
Murlished By james p.harrison&co
_______ Atlanta ,ga, SB
SUBSCRIPTION: 2.50 A YEAR.
				

